# Loading Density Influences the Tumor Cell Targeting and Signaling Inhibition Capabilities of Antibody Nanoconjugates

**DOI:** 10.1021/acsomega.5c13065

**Published:** 2026-01-27

**Authors:** George C. Kramarenko, Carolina Gomez Casas, Megan N. Dang, Nikos D. Demetriou, Emily S. Day

**Affiliations:** † Department of Biomedical Engineering, 5972 University of Delaware, Newark, DE 19716, United States; ‡ Department of Materials Science and Engineering, 5972 University of Delaware, Newark, DE 19716, United States; § Cawley Center for Translational Cancer Research, Helen F. Graham Cancer Center and Research Institute, Newark, DE 19713, United States

## Abstract

Triple-negative breast cancer (TNBC) is the most aggressive breast cancer subtype and accounts for up to 20% of all breast cancers. Since conventional chemotherapy and radiotherapy are ineffective against TNBC, nanoparticle-based medicines are being investigated as a potentially superior treatment option. Of such platforms, antibody–nanoparticle conjugates have been shown to precisely target diseased cells through selective antigen binding and to regulate oncogenic cellular signaling by blocking ligand activation of the targeted receptor. For example, silica core-gold shell “nanoshells” (NS) conjugated to Frizzled7 (FZD7) antibodies can preferentially bind TNBC cells to suppress Wnt signaling and inhibit disease progression. To improve understanding of antibody nanoconjugate structure/function relationships, in this study, we evaluated the influence of antibody loading density on the ability of FZD7-NS conjugates to bind TNBC cells, suppress Wnt signaling, and inhibit oncogenic cell behavior. We found that a lower antibody loading density of ∼60 antibodies per NS provided increased TNBC cellular binding and enhanced therapeutic efficacy compared to a higher antibody loading of ∼170 antibodies per NS. Specifically, the low-density FZD7-NS exhibited ∼2× greater binding avidity to MDA-MB-231 human TNBC cells than high-density FZD7-NS, yielding more robust inhibition of several Wnt target genes, as measured by RT-qPCR. Congruently, tumor spheroids formed from MDA-MB-231 cells that were pretreated with low-density FZD7-NS had significantly reduced area, metabolic activity, and cell number compared to those treated with high-density FZD7-NS. These results emphasize the importance of determining the appropriate surface ligand density when designing antibody–nanoparticle conjugates for therapeutic utility.

## Graphical abstract

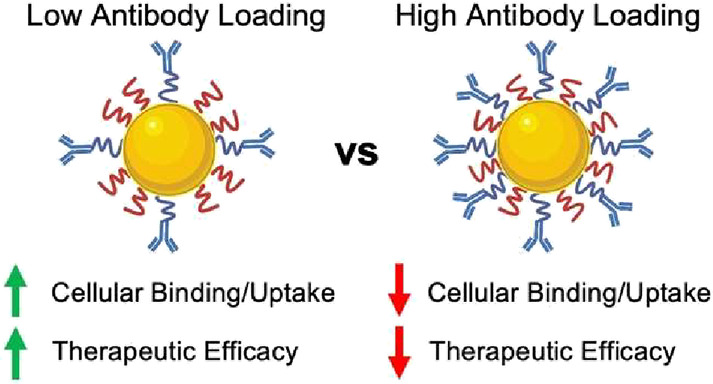

## 1 Introduction

Triple-negative breast cancer (TNBC) accounts for approximately 20% of all breast cancer cases and is associated with high mortality rates and poor patient outcomes.^1^ Unlike other subtypes of breast cancer, TNBC lacks all three breast cancer receptors of estrogen receptor, progesterone receptor, and human epidermal growth factor receptor 2 (HER2), which makes it unsusceptible to most targeted therapies that utilize these receptors for cellular binding.^2,3^ Standard treatment for TNBC includes surgery, radiation, and chemotherapy, which are often debilitating and can lead to severe off-target effects with associated morbidity.^4,5^ The lack of safe and effective treatments for TNBC make it critical to develop novel targeted therapies to better manage this aggressive disease.

One promising strategy to target and treat TNBC is to exploit the Wnt signaling pathway that is upregulated in TNBC cells, resulting in an abundance of FZD7 transmembrane receptors on the cell surface.^6–8^ When extracellular Wnt ligands bind these FZD7 receptors, it initiates nuclear translocation of intracellular β-catenin proteins, which then activate transcription of downstream genes that promote cancer cell proliferation, migration, invasion, and other oncogenic behaviors (Scheme 1).^9,10^ Blocking Wnt ligand binding to FZD7 receptors to suppress this signaling cascade may limit TNBC progression.^3,11^ Antibodies that can bind FZD7 antigens with high selectivity are particularly promising tools to achieve this signal cascade interference.^2,12,13^ However, freely delivered antibodies are therapeutically limited due to low binding affinities and rapid rates of clearance.^13–15^ Antibody-nanoparticle (NP) conjugates are a promising alternative because they can exploit multivalency to increase binding avidity.^13^ Whereas free antibodies exhibit 1:1 receptor binding, antibody-NP conjugates can engage multiple receptors simultaneously, which increases cellular binding strength and bioactivity. Antibody-NP conjugates can also be optimized through tuning NP size, composition, surface chemistry, and other characteristics.^16,17^

**Scheme 1:**
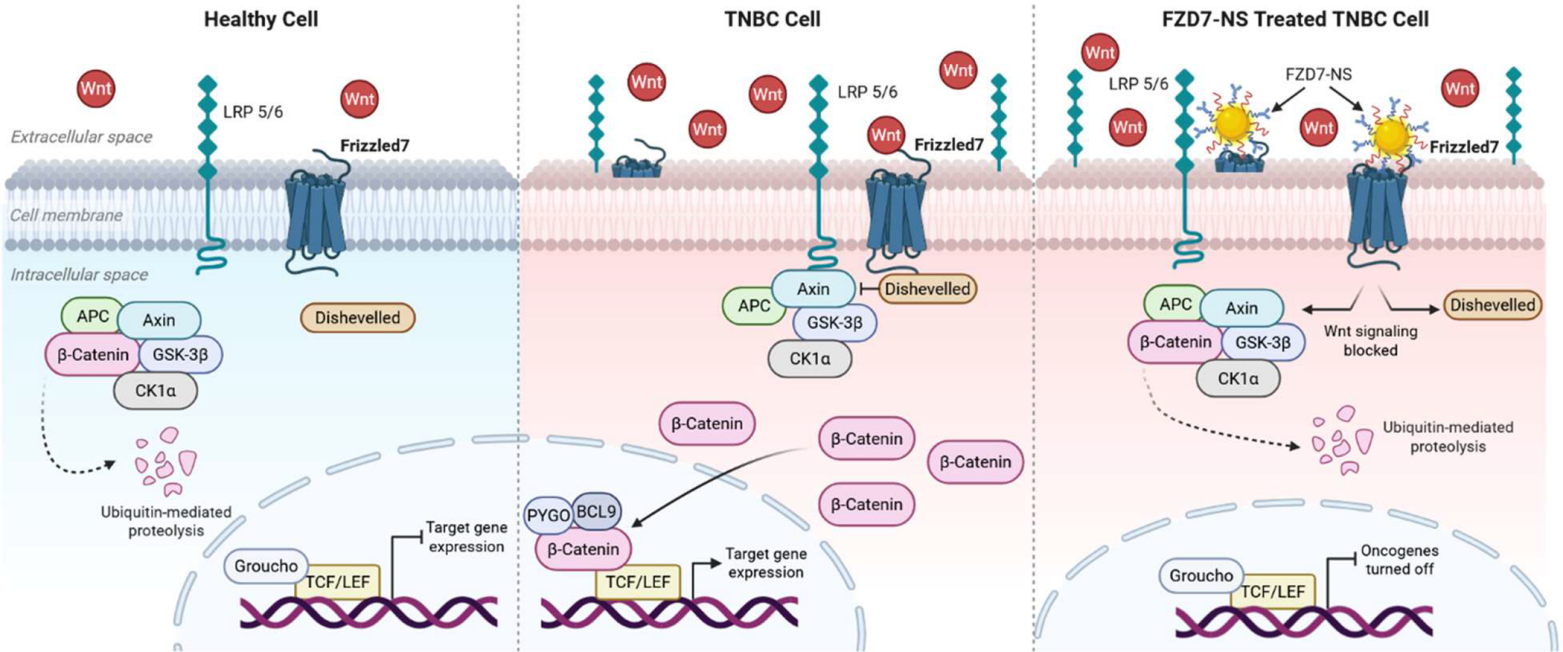
Illustration of Wnt Signaling Pathway in Noncancerous Cells (Left) Compared to TNBC Cells (Center) and How Antibody Nanoconjugates Can Competitively Bind Upregulated FZD7 Receptors on TNBC Cells to Suppress Wnt Signaling Activity (Right)^a^

a (Left) In healthy noncancerous cells, a destruction complex made up of Axin, adenomatous polyposis coli (APC), glycogen synthase kinase-3β (GSK-3β), and casein kinase 1 alpha (CK1α) continuously binds and phosphorylates β-catenin, leading to its degradation. (Center) In TNBC, Wnt ligand binding to overexpressed FZD7 receptors triggers recruitment of dishevelled, which inhibits the destruction complex to allow β-catenin to accumulate in the cytoplasm. β-catenin then translocates to the nucleus, where it associates with TCF/LEF proteins to activate the transcription of Wnt target genes that enable oncogenesis. (Right) FZD7-NS conjugates compete with Wnt ligands to effectively bind and block FZD7 receptors and inhibit the Wnt signaling pathway in TNBC. Adapted from “Wnt Signaling Pathway Activation and Inhibition,” by BioRender.com (2023). Retrieved from https://app.biorender.com/biorender-templates. Created in BioRender. Kramarenko, G. (2026) https://BioRender.com/7qgl7iu.

We have previously shown that FZD7 antibody-NP conjugates based on a “nanoshell” (NS) core can effectively block Wnt ligand/FZD7 receptor interactions to suppress Wnt signaling and oncogenic behavior in TNBC cells (Scheme 1).^13–15^ NS are beneficial as antibody carriers because they offer simple bioconjugation through gold/thiol bonding and have unique optical properties for ease of tracking.^18,19^ While our prior studies show FZD7-NS conjugates are effective in vitro and in vivo,^13–15^ an unanswered question is the influence of antibody loading density on cellular binding and treatment efficacy. Since antibody loading density may impact bioactivity,^20^ this investigation aimed to develop FZD7-NS with low or high antibody loading densities and evaluate their interactions with TNBC cells.

Previous research investigating the impact of antibody loading density has revealed two particular patterns for cellular uptake or tumor accumulation: optimum ligand density at a maximum or optimum ligand density at plateau.^20–25^ The first result indicates that beyond a certain ligand density, cellular uptake will decrease with higher concentrations of antibodies.^21,23,25–27^ Alternatively, the second outcome indicates no reduction in uptake after reaching a certain loading density.^22–25,27,28^ These varying outcomes indicate more work is needed to understand structure/function relationships of antibody-NP conjugates. Accordingly, this study aimed to understand the effect of antibody loading density on the ability of FZD7-NS conjugates to bind TNBC cells and inhibit intracellular Wnt signaling.

## 2 Experimental Section

### 2.1 Synthesis of Silica Core/Gold Shell NS

NS were synthesized by the method of Oldenburg et al.^29^ Briefly, 3–4 nm diameter gold colloid was made by adding 6.75 mL 29.7 mM hydrogen tetrachloroaurate (III) hydrate (HAuCl_4_) (VWR) to a beaker containing 180 g Milli-Q water, 4 mL of tetrakis­(hydroxymethyl)­phosphonium chloride (THPC) (VWR) (prepared by mixing 400 μL of the THPC stock with 33 g of Milli-Q water), and 1.2 mL of 1 M sodium hydroxide (Fisher Scientific) while stirring. The gold colloid was removed from the stir plate immediately and “aged” approximately 2 weeks at 4 °C. Next, 30 mL of gold colloid was mixed with 1 mL of 1 M sodium chloride (NaCl) and 180 μL of 120 nm silica spheres that were functionalized with 3-aminopropyltriethoxysilane (Nanocomposix); this mixture rocked for 3–5 days at room temperature to form “seed” nanoparticles. The seed nanoparticles were then centrifuged (25 min at 1650 g) and resuspended in Milli-Q water at an optical density (OD) of 0.1 at 530 nm. Following this, different volumes of seed (100–500 μL) were mixed with 1 mL of a growth solution (made in advance by diluting 12 mL 29 mM HAuCl_4_ and 200 mg potassium chloride in 800 mL Milli-Q water). Next, 10 μL of 37% formaldehyde (VWR) was quickly added and samples were vortexed rapidly to create complete gold shells around the silica cores. The ratio of seed, growth solution, and formaldehyde that produced NS with a maximum extinction of ∼810 nm as measured with a Cary60 UV–visible spectrophotometer was then linearly scaled up to produce a larger batch of NS. The synthesized NS were purified by centrifugation (500 g, 5 min, thrice) with probe sonication performed after each wash step to disperse the NS in Milli-Q water. The NS were stored at ∼6 × 10^9^ NS mL^–1^ (OD^810 nm^ = 2) at 4 °C until use.

### 2.2 Attachment of FZD7 Antibodies to Heterobifunctional Poly­(ethylene glycol) Linkers

Rabbit antihuman FZD7 antibodies (LSBio) were ordered at 1 mg mL^–1^. FZD7 antibodies were diluted in 1X PBS at 10X the original volume and purified via centrifugal filtration at 8 °C, 4200 rpm for 30 min using 10 kDa centrifugal filtration tubes (Amicon). After purification, antibodies were resuspended to the original stock volume in 1X PBS and combined with 5 kDa orthopyridyl disulfide-poly­(ethylene glycol)-succinimidyl valerate (OPSS-PEG-SVA, Laysan Bio) in 100 mM sodium bicarbonate at a 2:1 PEG:FZD7 molar ratio. The mixture was rocked overnight at 4 °C to form PEGylated antibodies (OPSS-PEG-FZD7), which were then aliquoted and stored at −20 °C until ready to use for NS conjugation.

### 2.3 Antibody and Methoxy-PEG-Thiol Conjugation to NS

NS diluted in Milli-Q water at OD^810 nm^ = 1.5 (4.5 × 10^9^ NS mL^–1^) were reacted with OPSS-PEG-FZD7 at a ratio of 400 antibodies/NS for the low loading condition or 1000 antibodies/NS for the high loading condition and rocked for 4 h at 4 °C. After this, the antibody-NS conjugates were bath sonicated and 5 kDa methoxy-poly­(ethylene glycol)-thiol (mPEG-SH, Laysan) was added at a final concentration of 10 μM and samples rocked at 4 °C overnight. Control nanoparticles without antibodies were prepared by adding only mPEG-SH to NS to form PEG-NS. The FZD7-NS and PEG-NS were separated from unbound OPSS-PEG-FZD7 and mPEG-SH by centrifuging at 500 g for 15 min once, followed by two 10 min centrifugation steps at 500 g, all in LoBIND low protein binding tubes (Fisher Scientific), with the unbound molecules discarded with the supernatant. The final purified FZD7-NS and PEG-NS were suspended in 1X PBS in a volume 100X less than the starting NS volume and stored at 4 °C until use. Concentrations of NS were determined by Beer’s Law based on the extinction maximum at ∼810 nm measured using on a Cary 60 UV–visible spectrophotometer.

### 2.4 Physicochemical Characterization of NS

The hydrodynamic diameter and zeta potential of bare NS, PEG-NS, and FZD7-NS were measured using an Anton Paar Litesizer dynamic light scattering (DLS) instrument. For this, NS samples were prepared at 3 × 10^9^ NS mL^–1^ (OD^810 nm^ = 1) in Litesizer cuvettes (for hydrodynamic diameter measurements) and Omega Cuvettes (for zeta potential measurements). DLS replicates had 100 runs each while zeta potential measurements included 1000 runs in replicates of 3. Plots of correlation function and size distribution by intensity for representative samples are provided in Supplemental Figure S1. A Thermo Scientific Talos L120C Transmission Electron Microscope (TEM) was used to visualize the morphology of each NS type. TEM images were obtained with support from the Delaware Bioimaging Center. Samples at 3 × 10^9^ NS mL^–1^ (OD^810 nm^ = 1) were added in small volumes to hydrophilic carbon support films with copper grids at 400 mesh, stained with 2% uranyl acetate, and dried prior to imaging.

### 2.5 Quantification of Antibody Loading on NS

Antibody loading on NS was quantified using an established enzyme-linked immunosorbent assay (ELISA).^30^ Centrifuge tubes were blocked with 3% w/v bovine serum albumin in 1X PBS (PBSA) via rocking for 30 min at room temperature. Control PEG-NS and FZD7-NS were prepared at OD^810 nm^ = 1 and added to the precoated tubes. Meanwhile, a solution of secondary horseradish peroxidase antirabbit IgG (HRP-AR) (SeraCare Life Sciences) was prepared and added to NS at an end concentration of 10 μg mL^–1^. The NS samples were vortexed and incubated for 1 h at room temperature protected from light. After this, NS were centrifuged at 1500 g for 5 min at room temperature, while 90% of the supernatant was removed. Samples were resuspended in the volume of PBSA removed and NS were dispersed by bath sonication. After repeating these purification steps a total of 3 times, the samples were resuspended in the original volumes of PBSA. Next, a portion of each sample was removed to be used as the positive measurement for antibody quantification, while another portion was centrifuged for a final time under the previous purification conditions to utilize the supernatant as the negative measurement. The positive and negative samples were added to a 96-well plate in triplicate at a 10X dilution in 1X PBS and measured against a standard curve made from the stock HRP-AR solution. Next, 3,3′,5,5′- tetramethylbenzidine (TMB Core, BioRad) was added to the standard curve and samples at a 5X volume and the plate was incubated at room temperature for 1 min. Then, 2 M sulfuric acid at a 1:1 volume of TMB core was added to all the wells to stop the reaction. A Biotek Synergy H1Microplate Reader was used to read the absorbance at 450 nm. Analysis consisted of subtracting negative sample measurements from positive measurements in order to eliminate background signal and the data from NS samples was plotted against the standard curve values. The number of antibodies per NS was calculated by dividing the measured number of antibodies by the number of NS in the sample.

### 2.6 Cell Culture and Experimental Setup for NS Treatment

MDA-MB-231 human TNBC cells (American Type Culture Collection, ATCC) were cultured in Dulbecco’s Modified Eagle Medium (DMEM; Fisher Scientific) supplemented with 10% fetal bovine serum (FBS; Gemini Bio) and 1% penicillin-streptomycin (VWR). MCF-10A noncancerous breast epithelial cells were used as a control for microscopy studies and cultured in 1:1 DMEM and F12 base medium supplemented with 5% FBS (Gemini Bio), 1% penicillin-streptomycin (VWR), 10 μg mL^–1^ insulin (ThermoFisher), 0.5 μg mL^–1^ hydrocortisone (Sigma), 50 μg mL^–1^ bovine pituitary extract (ThermoFisher), 20 ng mL^–1^ epidermal growth factor (ThermoFisher), and 100 ng mL^–1^ cholera toxin (Sigma). Cells were cultured in T75 cell culture flasks (VWR) at 37 °C in a 5% CO_2_ humidified environment. Cells were passaged or plated upon reaching ∼80% confluency and were lifted from the flask using 0.25% trypsin-EDTA (VWR). Cells were counted using a hemocytometer and either transferred into a new T75 flask or into chambered coverglass or well plates as appropriate for the experiment. After overnight culture, the cells were incubated with complete media or with PEG-NS, Low FZD7-NS, or High FZD7-NS diluted in media at 3 × 10^9^ NPs mL^–1^ (OD^810 nm^ = 1) or 6 × 10^9^ NPs mL^–1^ (OD^810 nm^ = 2) for varying periods of time as specified in each experiment. For antibody-NS conjugates, treatment at OD^810 nm^ = 1 corresponds to ∼0.8 nM FZD7 antibodies for High FZD7-NS and ∼0.3 nM FZD7 antibodies for Low FZD7-NS.

### 2.7 Multiphoton Microscopy of NS Binding to TNBC Cells and Measurement of Binding Avidity

NS binding to MDA-MB-231 TNBC cells or MCF10A noncancerous breast epithelial cells (Supplemental Figures S2 and S3) was assessed using multiphoton microscopy, as NS exhibit two-photon induced photoluminescence in the range of 450–600 nm after excitation with femtosecond-pulsed 800 nm light.^31^ Our previous work showed that MDA-MB-231 cells overexpress FZD7 receptors relative to MCF10A cells.^13^ Cells were seeded in Nunc Lab-Tek #1 8-well chamber slides (ThermoFisher) at 250,000 cells per well and allowed to adhere for at least 16 h incubating at 37 °C, 5% CO_2_. The following day, cell culture media was aspirated, and cells were treated with PEG-NS, Low FZD7-NS, or High FZD7-NS at OD^810 nm^ = 2 in complete media for 4 h. After NS incubation, the media was removed, and cells were washed 3 times with warm Dulbecco’s 1X PBS (DPBS, ThermoFisher). Cells were then fixed with 4% paraformaldehyde in 1X DPBS for 10 min at room temperature and neutralized with 1X DPBS afterward. Cell membranes were stained using CellVue Claret Far Red Membrane Dye (Sigma-Aldrich) for 5 min at room temperature. Next, 1% PBSA was added to cells to neutralize the dye, and replaced with fresh 1X DPBS after aspiration. A Zeiss LSM 880 Multiphoton Microscope with a 25*x*/0.8 NA water objective was used to image slides. The slides containing NS were excited by the multiphoton laser at λ_excitation_ = 800 nm with a pinhole of 1.57 AU and detection range λ_emission_ of 400–550 nm. Cell membranes stained with CellVue Claret Red were imaged at λ_excitation_ = 655 nm and λ_emission_ of 675 nm.

The binding avidity of Low FZD7-NS and High FZD7-NS to MDA-MB-231 cells was evaluated as follows. MDA-MB-231 cells were seeded at 55,000 cells/well in a 96 well plate and incubated for 16–20 h to allow cell adhesion. The cells were then fixed with 4% formaldehyde (Sigma-Aldrich) in 1X PBS and incubated 15 min at room temperature. The formaldehyde was neutralized with 1X PBS, and wells were washed with 1X PBS three more times. Subsequently, cells were blocked for endogenous peroxidase activity with 3% hydrogen peroxide (BDH) in 1X PBS for 10 min. After removing the hydrogen peroxide, 100 μL of 3% PBSA was added to each well and incubated for 2 h. A range of NS concentrations for both Low FZD7-NS and High FZD7-NS were prepared in Milli-Q water, with the OD^810 nm^ ranging from 15 to 0.25, which is equivalent to 4.6 × 10^10^ NS mL^–1^ to 7.6 × 10^8^ NS mL^–1^. These NS concentrations were added to the samples (with three replicates for each concentration) and incubated at room temperature for 1.5 h. NS were then removed and samples washed 3X with 1% PBST (1% PBSA with 0.01% Tween-20, Sigma-Aldrich) for 10 min each. After this, 2.5 μg/mL HRP-antirabbit secondary antibody (400X dilution) in 3% PBSA was added and incubated for 1 h. Samples were washed 3X with 1% PBST for 15 min each. The 1% PBST was aspirated, and TMB was added to samples and incubated for 30 s to 1 min; following this, 2 M sulfuric acid was added to stop the reaction. A Synergy H1 plate reader (BioTek) was used to measure absorbance at 450 nm. Data were fit to a modified Langmuir isotherm model to determine the effective dissociation constant (*K*_D_).^32^ The equation used was $D=\frac{{\mathrm{Ab}}_{\mathrm{conc}}}{K_{D}+{\mathrm{Ab}}_{\mathrm{conc}}}$ , where Ab_conc_ corresponds to the antibody concentration added to the cells. Moreover, the optical density (OD) at each antibody concentration was normalized with the following equation $D=\frac{{\mathrm{OD}}_{\mathrm{raw}}-{\mathrm{OD}}_{\mathrm{back}}}{{\mathrm{OD}}_{\mathrm{high}}-{\mathrm{OD}}_{\mathrm{back}}}$ , where OD_raw_ represents the initial optical density reading (prior to any calculation), OD_back_ is the background signal which corresponds to the cells that were treated with no primary antibody and treated with secondary antibody, and OD_high_ is the highest (saturated) signal. The latter equations were applied to the modified Langmuir isotherm model in JMP statistical software.

### 2.8 Flow Cytometry Analysis of NS Interaction with TNBC Cells

To assess NS interactions with MDA-MB-231 cells by flow cytometry, NS were conjugated with Cy5-PEG-SH in addition to mPEG-SH. The NS were conjugated with 1 μM Cy5-PEG-SH and 9 μM mPEG-SH to ensure both equal PEGylation additions and Cy5 signal across groups (Supplemental Figure S4). After Cy5-conjugated PEG-NS, Low FZD7-NS, and High FZD7-NS were synthesized, purified, and characterized for their fluorescence, hydrodynamic diameter, and zeta potential (Supplemental Figure S4), they were added to MDA-MB-231 cells (seeded in 12-well plates at 50,000 cells/well and adhered for ∼16 h before treatment) at OD^810 nm^ = 1 or OD^810 nm^ = 2 in complete media. The cells were incubated with NS for 4 or 24 h at 37 °C. At these times, the culture media was removed and cells were washed with 1X DPBS 3 times to remove noninternalized/unbound NS. After washing, cells were extracted using trypsin, neutralized in 1X DPBS, and transferred to Eppendorf tubes for flow cytometry. Cell suspensions were analyzed using an Acea Novocyte 2060 Flow Cytometer with the APC channel (excitation = 640 nm; emission = 675/30 nm). Density plots showing forward and side scatter data were used to make a primary gate to indicate the population of cells and to exclude debris. When processing the data, the median fluorescence intensity (MFI) was averaged in triplicate and NS treatment groups were normalized to the nontreated group.

### 2.9 RT-qPCR Analysis of Messenger RNA (mRNA) Expression of Wnt-Related Genes

Real-time reverse transcription quantitative polymerase chain reaction (RT-qPCR) was used to measure the impact of NS treatment on mRNA expression of relevant Wnt-associated genes. MDA-MB-231 cells were plated at 100,000 cells/well in 6-well plates prior to NS treatment. Cells were treated with no NS, PEG-NS, Low FZD7-NS, or High FZD7-NS at OD^810 nm^ = 2 and incubated for 24 h at 37 °C. After NS incubation, total RNA was extracted using the Isolate II RNA Mini Kit (Bioline). Modifications to the protocol include omitting the β-mercaptoethanol addition. RNA concentration was determined by reading the sample absorbance at 260 nm using a Take3 Plate on a Synergy H1 plate reader. RT-qPCR was then performed using SensiFAST SYBR One-Step Master Mix (Thomas Scientific) on a LightCycler96 (Roche). The RT-qPCR settings for SYBR Green I included quant factor at 20.00, melt factor at 1.20, and integration time at 1 s with an overall Dynamic Integration Time (s). The run profile included two preincubation steps for 1 cycle, which was followed by a 45 s cycle 3-step amplification, and this was followed by 1 cycle of melting. The Step setting was for 1200 s at 48 °C. Gene expression was normalized to that of β-glucuronidase (GUSB) and further normalized to nontreated group. Primer sequences are listed in Table S1. These experiments were performed in triplicate. RT-qPCR data was analyzed by the delta delta CT method and differences in mean mRNA expression between groups were determined by one-way ANOVA with posthoc Tukey.

### 2.10 Spheroid Formation in Response to NS Treatment

To examine the self-renewal capabilities of TNBC cells treated with FZD7-NS, a spheroid formation assay was employed. MDA-MB-231 cells were plated at 100,000 cells/well in 6-well plates and treated with no NS or with PEG-NS, Low FZD7-NS, or High FZD7-NS at OD^810 nm^ = 2 for 24 h at 37 °C. After incubation, cells were trypsinized and resuspended in MammoCult Basal Medium (StemCell) supplemented with MammoCult Proliferation Supplement, 4 μg mL^–1^ heparin (StemCell), and 0.48 μg mL^–1^ hydrocortisone (StemCell). The cells were reseeded at 5000 cells in low adhesion U-bottom 96 well plates (BrandTech) and incubated at 37 °C to form spheroids over 7 days. Brightfield imaging was performed every 3 days using an Axioobserver Z1 Inverted Fluorescent Microscope (Zeiss) with a 10X objective. Spheroid area on Day 1, 4, and 7 were measured using ImageJ.

After the seventh day of spheroid formation, the therapeutic efficacy of NS treatments was assessed by measuring metabolic activity via alamarBlue assay and by quantifying the number of cells in spheroids using flow cytometry. For the metabolic activity assay, spheroids were treated with 1X alamarBlue (ThermoFisher) per the manufacturer’s instructions for 24 h while incubated at 37 °C. Following this, fluorescence was measured using a Synergy H1 plate reader (BioTek) with excitation/emission of 560/590 nm. After measuring metabolic activity, spheroids were prepared for flow cytometry to acquire a cell count. Spheroids were washed twice with 1X DPBS to remove metabolic activity reagent and dissociated by treating with 0.25% trypsin for 15 min. Spheroids became dissociated after gentle pipetting/vortexing and cell suspensions were neutralized with 1X DPBS and placed in 1.5 mL Eppendorf tubes. Cell suspensions were centrifuged at 300 rcf at room temperature for 5 min and resuspended in fresh 1X DPBS. Cell suspensions were analyzed for cell count using an Acea Novocyte 2060 flow cytometer (Supplemental Figure S5). Density plots illustrating forward scatter height (FSC-H) and side scatter height (SSC-H) were used to make a primary gate for the cell population. A second plot was created to gate for singlet cells by plotting FSC-H vs forward scatter area (FSC-A). The number of events in this gate was used as the metric for total singlet cell count per spheroid. Both spheroid metabolic activity and cell counting experiments were performed in triplicate and differences between groups were analyzed by one-way ANOVA with posthoc Tukey.

## 3 Results and Discussion

### 3.1 Characterization of NS and NS Conjugates

NS were synthesized and modified with mPEG-SH alone (PEG-NS) or with low or high concentrations of FZD7 antibodies (Low FZD7-NS and High FZD7-NS) as described in the Experimental Section and depicted in Figure 1A. Prior to surface modification, Bare NS had a hydrodynamic diameter of 171.6 ± 2.0 nm and a zeta potential of – 33.2 ± 2.2 mV (Figure 1B and Supplemental Figure S1B). The addition of mPEG-SH on PEG-NS allowed for a size increase to 189.8 ± 1.6 nm and charge neutralization to −5.4 ± 1.0 mV. Following attachment of OPSS-PEG-FZD7 and mPEG-SH, the Low FZD7-NS and High FZD7-NS had diameters of 199.0 ± 2.4 nm and 206.2 ± 4.7 nm, respectively, and zeta potentials of −7.9 ± 0.7 mV and −8.9 ± 1.1 mV, respectively. Antibody loading density was measured by ELISA to be 62 ± 5 antibodies per NS for Low FZD7-NS and 171 ± 5 antibodies per NS for High FZD7-NS (Figure 1C). These loading densities correspond to 670 antibodies per μm^2^ surface area for Low FZD7-NS and 1848 antibodies per μm^2^ for High FZD7-NS. The conjugation efficiency was ∼16 and ∼17% for Low FZD7-NS and High FZD7-NS, respectively, compared to the number of antibodies added initially. Bare NS and NS conjugates were spherical when imaged using TEM (Figure 1D). Additionally, all NS types were stable in storage over a 72-h time frame based on repeated measurements of hydrodynamic diameter, zeta potential, and antibody loading density (Supplemental Figure S6).

**Figure 1:**
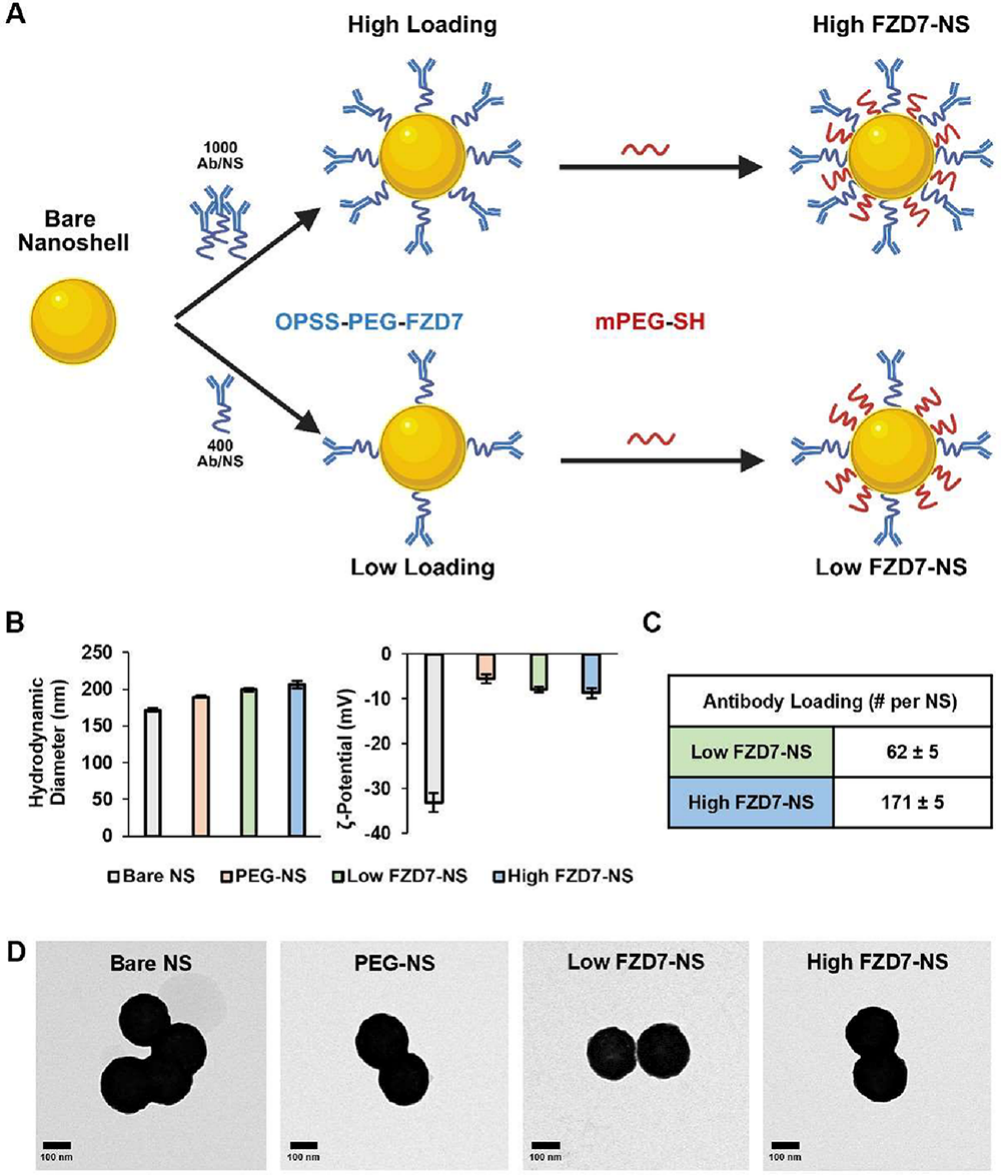
Characterization of bare NS and NS conjugates. (A) Scheme depicting the attachment of OPSS-PEG-FZD7 and mPEG-SH to NS. Created in BioRender. Kramarenko, G. (2026) https://BioRender.com/oml65ga. (B) Hydrodynamic diameter and zeta potential of bare NS compared to conjugated NS. (C) FZD7 antibody loading of low FZD7-NS and high FZD7-NS. (D) Transmission electron micrographs of bare NS, PEG-NS, low FZD7-NS, and high FZD7-NS. Data in panels B and C represent the mean ± standard error of *n* = 5 batches for each NP type.

### 3.2 FZD7 Antibody-Conjugated NS Effectively Bind TNBC Cells

There is higher expression of FZD7 receptors in MDA-MB-231 TNBC cells compared to MCF10A noncancerous breast epithelial cells.^10,13^ Accordingly, we assessed the ability of FZD7-NS and PEG-NS to bind each cell type (Figure 2A, Supplemental Figures S2 and S3). Two-photon microscopy was used to visualize NS in treated cells.^31^ As predicted, MCF10A breast epithelial cells had limited binding of all NS types (Supplemental Figure S3), while MDA-MB-231 cells had higher binding of FZD7-NS than PEG-NS (Figure 2B and Supplemental Figure S2). When comparing the FZD7-NS groups visually, there appears to be greater binding of Low FZD7-NS than High FZD7-NS to MDA-MB-231 cells.

**Figure 2:**
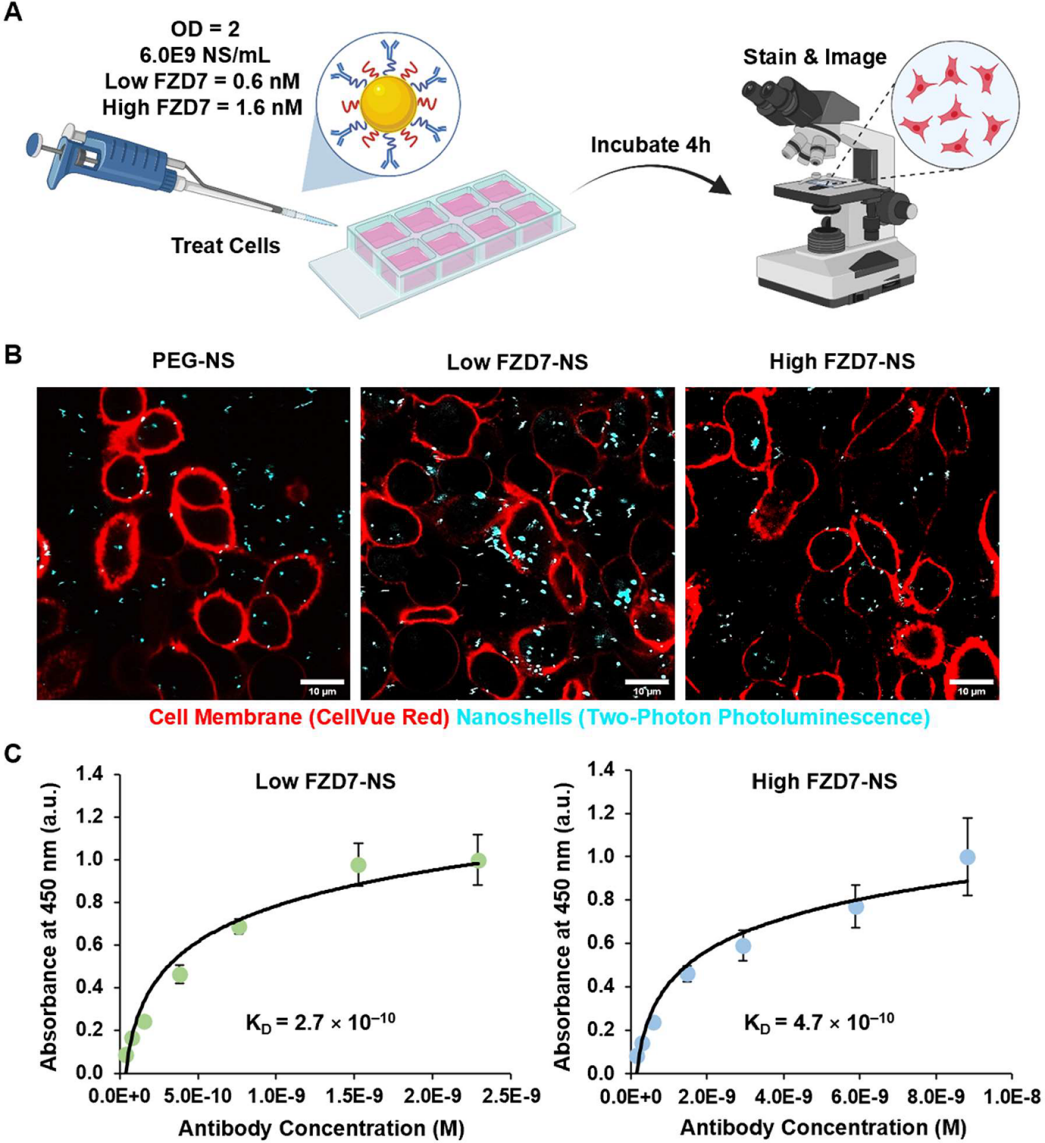
Analysis of NS conjugate binding to TNBC cells. (A) Scheme of NS treatment. Created in BioRender. Kramarenko, G. (2026) https://BioRender.com/930z9pw. (B) Multiphoton microscopy images of NS (blue) binding to MDA-MB-231 cells (red). Scale bars = 10 μm. (C) Effective dissociation constant for low FZD7-NS and high FZD7-NS to MDA-MB-231 cells. Data were fit to a modified Langmuir isotherm model (*n* = 3).

To quantitatively compare the binding of Low FZD7-NS and High FZD7-NS to TNBC cells, a binding avidity assay was employed as described by Puig et al. and Riley et al.^13,32^ This model describes the binding of a ligand (FZD7-NS in this case) to an immobilized antigen of fixed TNBC cells. We found an effective dissociation constant (*K*_D_) of 2.7 × 10^–10^ M for Low FZD7-NS and 4.7 × 10^–10^ M for High FZD7-NS (Figure 2C). To put this in context, we previously reported a *K*_D_ of 1.48 × 10^–8^ M for free FZD7 antibodies;^13^ hence, FZD7-NS conjugates have greatly increased binding to FZD7 receptors on TNBC cells compared to free antibodies. Comparing the *K*_D_ of the two NS conjugates indicates Low FZD7-NS have ∼2-fold increased binding avidity to FZD7 surface receptors of TNBC cells compared to High FZD7-NS. The representative binding curve for Low FZD7-NS reached saturation, indicated by the slowly plateauing plot at antibody concentrations beyond 1.5 × 10^–9^ M, while High FZD7-NS did not reach saturation. It should be noted, however, that the final data point for High FZD7-NS displays a large standard deviation, such that it is challenging to definitively conclude if saturation has been reached; repeating the assay with an additional data point at a higher concentration may improve confidence in the result. Nevertheless, the difference in the calculated *K*_D_ suggests that when there is a higher antibody density on NS, there is reduced binding to the cells.^13^ We postulate that steric hindrance and altered orientation of antibodies on the NS surface occurs when the loading density is high, which results in reduced receptor–ligand interactions.

We further investigated cellular binding using flow cytometry (Figure 3A). NS were conjugated with Cy5-tagged mPEG-SH, and we confirmed all NS groups had equivalent Cy5 signal (Supplemental Figure S4). MDA-MB-231 cells were treated with NS at concentrations of OD^810 nm^ = 1 (Supplemental Figure S7) or OD^810 nm^ = 2 for 24 h (Figure 3B). At both concentrations, both FZD7-NS groups had significantly greater cellular uptake than PEG-NS, but there was only statistical significance between Low FZD7-NS and High FZD7-NS at OD^810 nm^ = 2, where Low FZD7-NS showed greater cellular uptake than High FZD7-NS (Figure 3B,C and Supplemental Figure S7). These findings are consistent with the cellular binding results (Figure 2) in that antibody conjugated NS showed greater accumulation in TNBC cells compared to PEG-NS, and a lower antibody loading density resulted in greater cell binding than higher antibody loading density.

**Figure 3:**
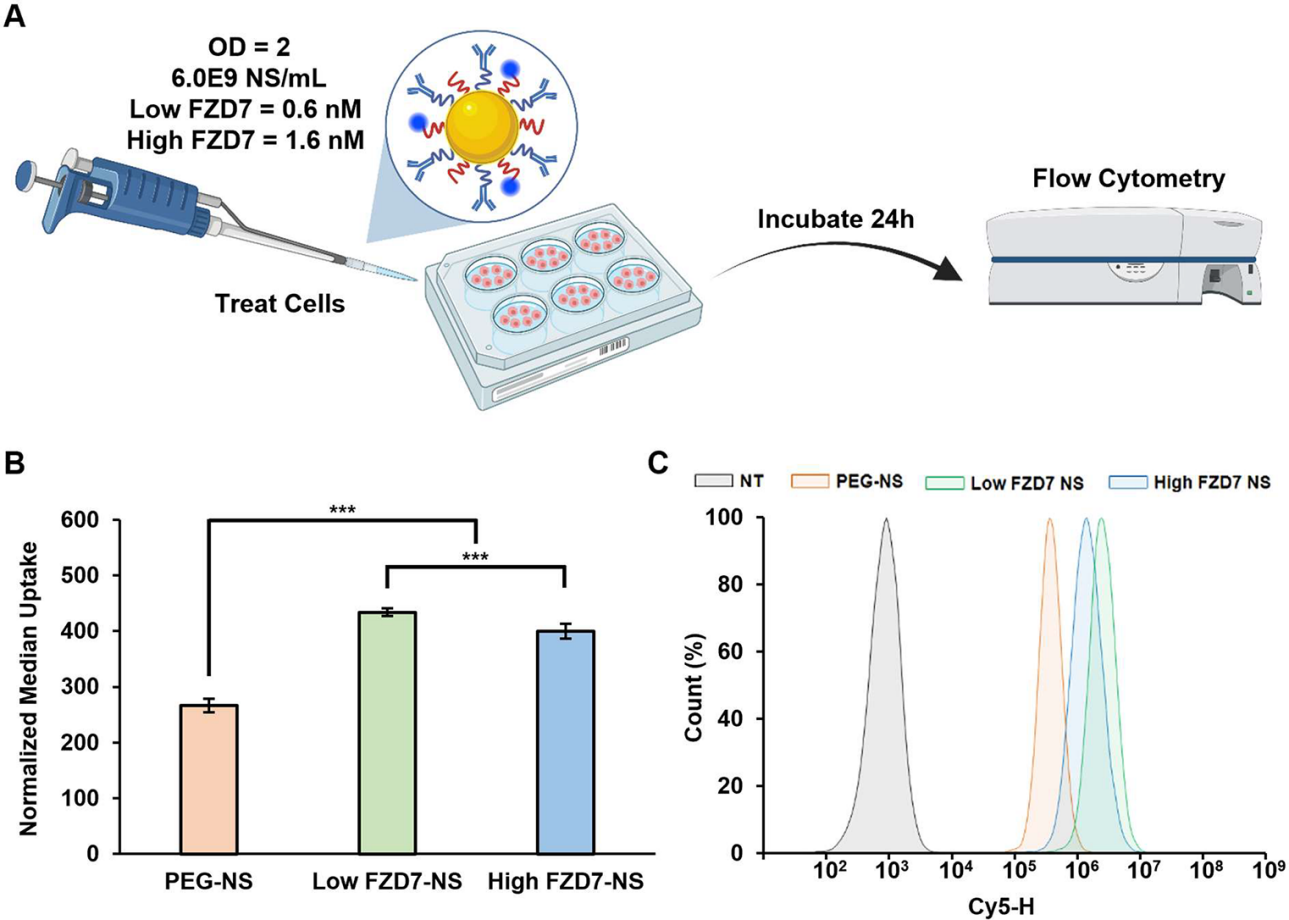
NS cellular interactions determined by flow cytometry. (A) Scheme of NS treatment. Created in BioRender. Kramarenko, G. (2026) https://BioRender.com/ndxx1lq. (B) Cellular uptake of Cy5-tagged NS after 24 h incubation quantified via flow cytometry. Data are normalized to nontreated cells and represent the mean ± standard error of the mean (*n* = 5). Statistical analysis by one-way ANOVA with post hoc Tukey; ***p < 0.001 between PEG-NS and both FZD7-NS groups and between low FZD7-NS and high FZD7-NS. (C) Representative flow cytometry histogram of NS cellular interactions at a concentration of OD = 2.

### 3.3 FZD7 Antibody–NS Conjugates Suppress Several Wnt Target Genes in TNBC Cells

To assess the effectiveness of FZD7-NS in regulating Wnt signaling, we evaluated the expression of several Wnt-associated genes at the mRNA level via RT-qPCR (Figure 4A). The major target gene of interest was β-catenin since this is the key mediator of the Wnt signaling pathway.^3,9^ We also examined several downstream genes that are involved in proliferation or stemness in TNBC, including Axin2, CD44, Nanog, KLFR4, and Oct4.^9,33,34^ After treating MDA-MB-231 cells for 24 h at OD^810 nm^ = 2, there was a significant reduction in mRNA expression of the evaluated genes when comparing FZD7-NS to both PEG-NS and the nontreated control (Figure 4B). Examining β-catenin expression, Low FZD7-NS and High FZD7-NS illustrated 2.7-fold and 1.5-fold greater knockdown compared to PEG-NS, respectively, and the difference between Low FZD7-NS and High FZD7-NS was statistically significant. Other analyzed genes indicated the same general trend in which there was increased mRNA suppression for Low FZD7-NS in comparison to High FZD7-NS, although the differences between these groups were not statistically significant at the 95% confidence level for genes downstream of β-catenin. PEG-NS had no significant effect on the expression of any evaluated gene compared to the untreated group, which confirms the observed reduction in FZD7-NS treated cells can be attributed to the nanoparticles’ ability to bind FZD7 receptors and lock them in Wnt ligand-unresponsive state.

**Figure 4:**
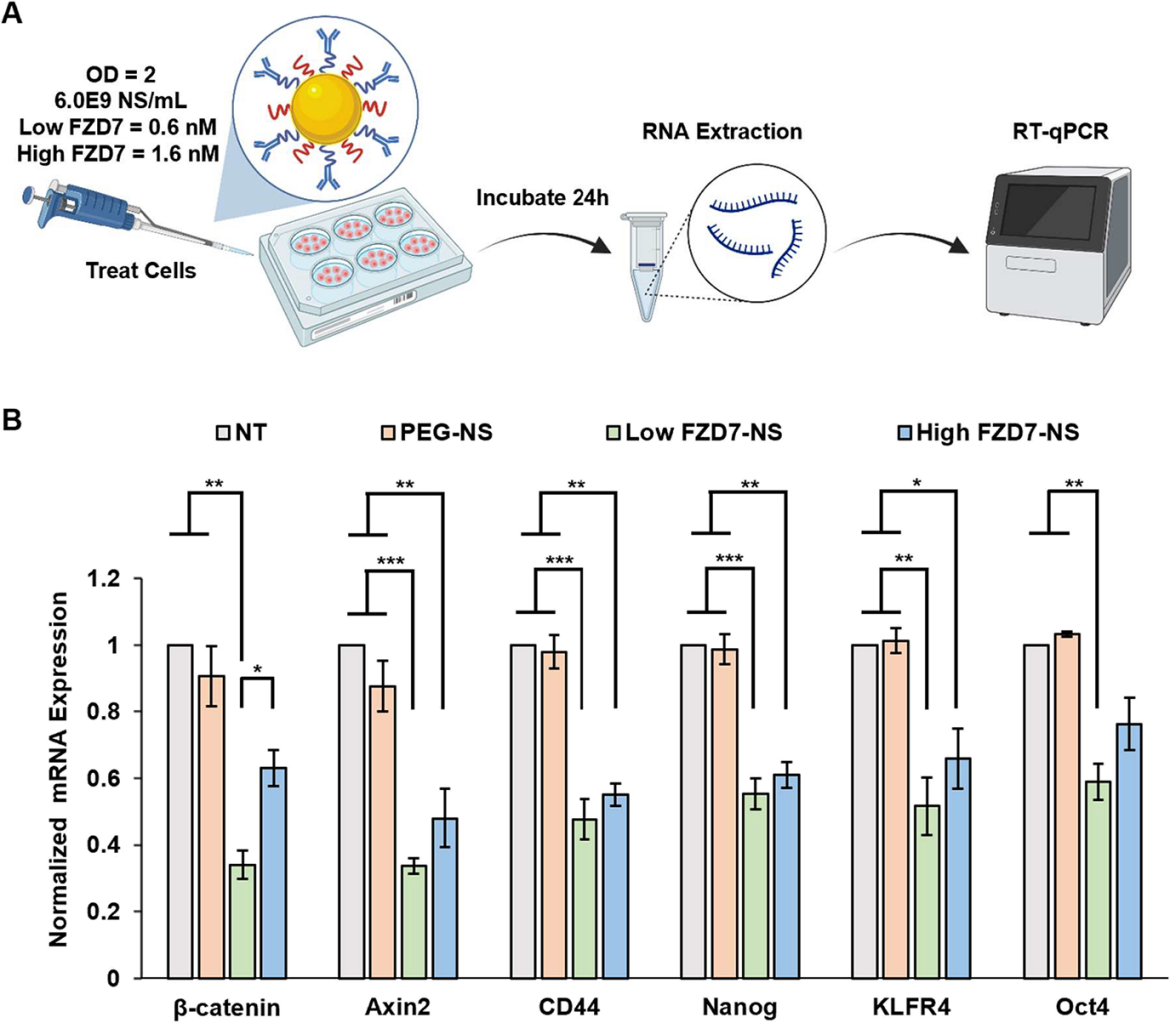
NS mediated gene regulation determined by RT-qPCR. (A) Scheme of treatment. Created in BioRender. Kramarenko, G. (2026) https://BioRender.com/7tut68k. (B) Relative mRNA expression of genes associated with the Wnt signaling pathway in TNBC cells. Data are normalized to the NT (nontreated) group for each gene and represent mean ± standard error (*n* = 3–4). Statistical analysis by one-way ANOVA with post hoc Tukey–Kramer; ***p < 0.001, **p < 0.01, *p < 0.05.

### 3.4 FZD7-NS Inhibit Spheroid Formation

The Wnt signaling pathway is closely related to cancer stemness, promoting cancer cell proliferation, tumor formation, and metastasis. Given that FZD7-NS can down-regulate several stemness genes (Figure 4), we wanted to determine if they could also inhibit TNBC cells’ ability to form 3D cancer spheroids. MDA-MB-231 cells were pretreated with OD^810 nm^ = 2 and OD^810 nm^ = 1 NS for 24 h and then reseeded into low-adhesion u-bottom well plates with spheroid promoting cell culture media (Figure 5A). Spheroids were allowed to grow up to 7 days and images were taken every 3 days to assess sphere formation (Figure 5B). Spheroids grew tightly and were compact forming dark regions of high cell counts in the center, where cells branching around the edges were lighter in color. Using spheroid images, the spheroid area over 7 days was measured in ImageJ for each treatment group (Figure 5C). All groups illustrated a linear growth in area with ∼3-fold size increase from Day 1 to Day 7. At both OD^810 nm^ = 1 and OD^810 nm^ = 2, Low FZD7-NS yielded spheroids with the smallest area at Day 7 (Figure 5D and Supplemental Figure S8). However, there were no statistically significant differences between treatment or control groups at OD^810 nm^ = 1, and at OD^810 nm^ = 2 the Low FZD7-NS were only statistically significant compared to the control groups of nontreated cells and PEG-NS.

**Figure 5:**
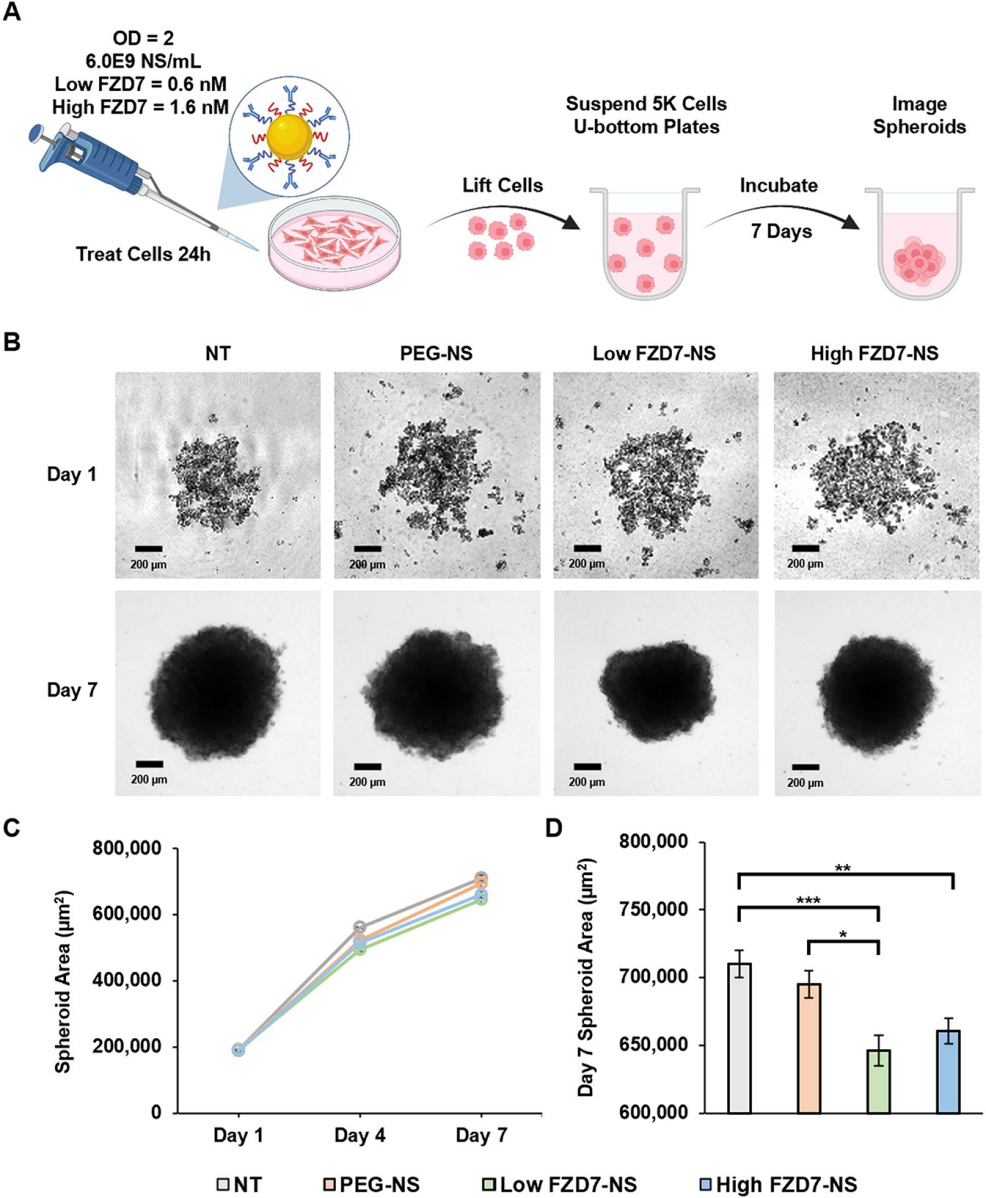
Spheroid formation after NS treatment at OD^810 nm^=2. (A) Scheme of spheroid formation assay. Created in BioRender. Kramarenko, G. (2026) https://BioRender.com/oe5z10k. (B) Representative brightfield images of formed spheroids after 7 days post 24 h NS treatment. Scale bar = 200 μm. (C) Spheroid area over time. (D) Spheroid area on day 7 across treatment types. Data show mean ± standard error (*n* = 3). Statistical analysis by one-way ANOVA with post hoc Tukey; ***p < 0.001,**p < 0.01, *p < 0.05.

To further examine the capability of FZD7-NS to hinder sphere formation, we assessed cell metabolic activity of spheroids after 7 days (Figure 6A and Supplemental Figure S9). Compared to nontreated spheroids, metabolic activity was reduced by ∼28% for Low FZD7-NS and ∼12% for High FZD7-NS at OD^810 nm^ = 2, while PEG-NS did not reduce metabolic activity (Figure 6B). The ∼16% greater inhibition for Low FZD7-NS versus High FZD7-NS was statistically significant.

**Figure 6:**
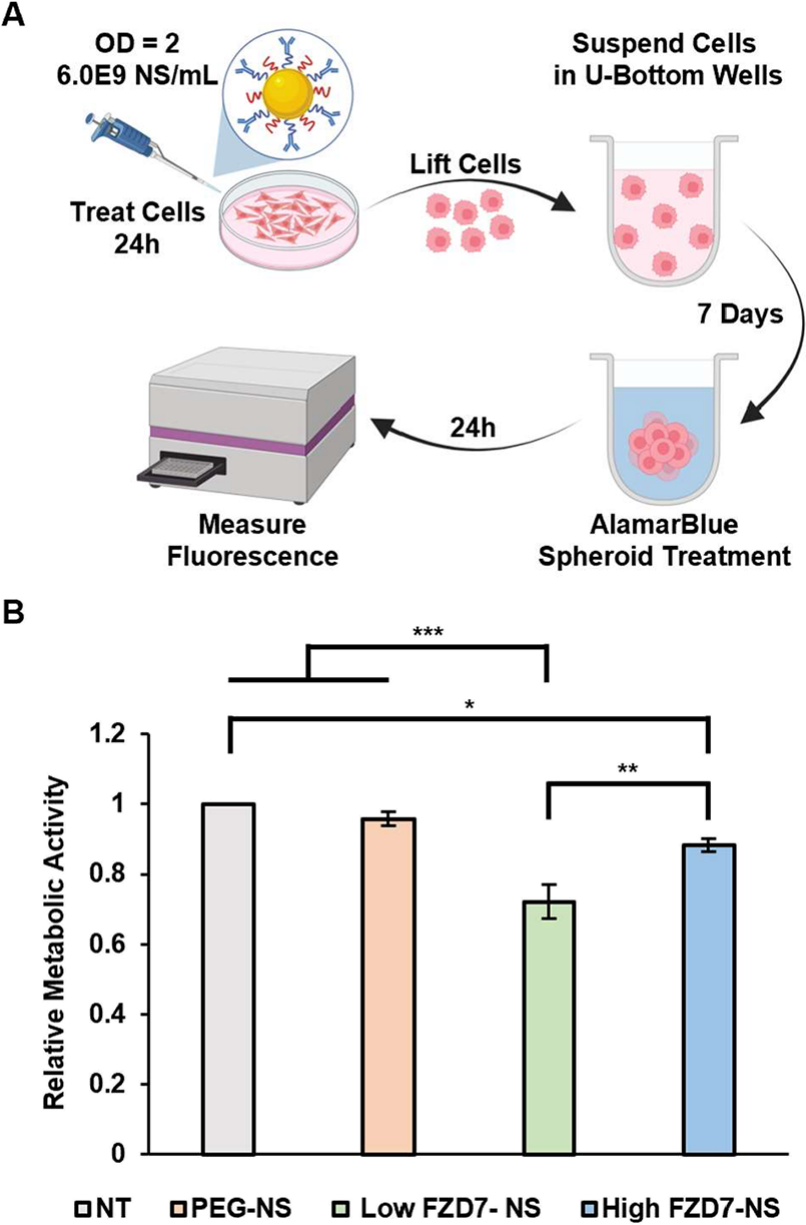
Spheroid metabolic activity in response to NS treatment. (A) Scheme of spheroid metabolic activity procedure. Created in BioRender. Kramarenko, G. (2026) https://BioRender.com/eb1rl35. At OD = 2, the corresponding antibody concentration is 0.6 nM for low FZD7-NS and 1.6 nM for high FZD7-NS. (B) Metabolic activity of spheroids measured by AlamarBlue assay 1 week after formation. Data are normalized to NT and represent mean ± standard error (*n* = 3). Statistical analysis by one-way ANOVA with post hoc Tukey; ***p < 0.001,**p < 0.01, *p < 0.05.

We further determined the impact of treatment on spheroids by counting cells with flow cytometry after spheroid dissociation (Figure 7A, Supplemental Figure S10). There was a great reduction in spheroid cell count by 48 and 30% for Low FZD7-NS and High FZD7-NS, respectively, at OD^810 nm^ = 2 compared to nontreated spheroids (Figure 7B). The 26% lower cell count for Low FZD7-NS versus High FZD7-NS was statistically significant. Similar impacts of treatment on spheroid metabolic activity and cell count were observed at OD^810 nm^ = 1, though to a lesser degree such that differences between groups were not significant (Supplemental Figure S10). Overall, the combined metabolic activity and cell count analysis of treated spheroids indicate Low FZD7-NS best inhibit spheroid formation compared to High FZD7-NS and the PEG-NS control.

**Figure 7:**
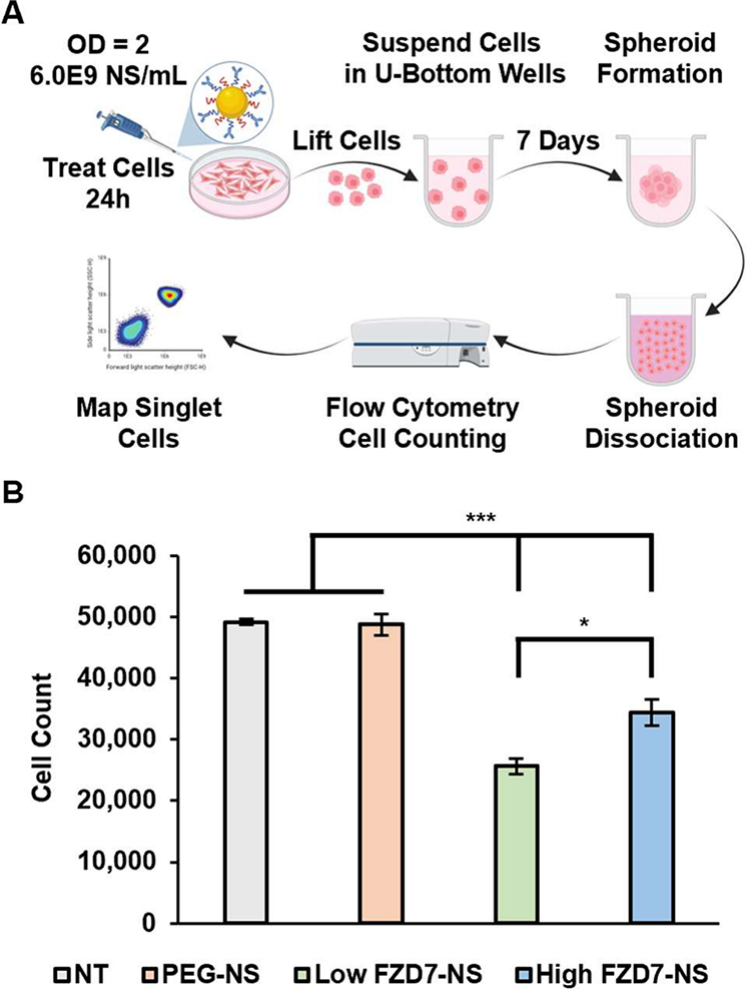
Spheroid growth inhibition in response to NS treatment. (A) Scheme of spheroid dissociation cell counting procedure. Created in BioRender. Kramarenko, G. (2026) https://BioRender.com/lcgbi6t. At OD = 2, the corresponding antibody concentration is 0.6 nM for low FZD7-NS and 1.6 nM for high FZD7-NS. (B) Cell counts of dissociated spheroids. Data represent mean ± standard error (*n* = 3). Statistical analysis by one-way ANOVA with post hoc Tukey; ***p < 0.001, *p < 0.05.

## 4 Conclusions

In this study, we developed FZD7-NS conjugates with two different antibody loading densities that are capable of binding TNBC cells in vitro and suppressing intracellular Wnt signaling. Our prior work has shown that FZD7-NS are therapeutically advantageous compared to free FZD7 antibodies or nontargeted NS;^13–15^ however, the effect of antibody loading density on cellular targeting and TNBC suppression remained unknown.

FZD7-NS were synthesized at low or high antibody loading densities and demonstrated stability over time (Figure 1). Multiphoton microscopy and binding avidity assays showed that FZD7-NS bind TNBC cells more efficiently than noncancerous cells and that Low FZD7-NS exhibit enhanced binding versus High FZD7-NS and PEG-NS (Figure 2, Supplemental Figures S2 and S3). The improved cellular interactions of Low FZD7-NS compared to High FZD7-NS were confirmed by flow cytometry using Cy5-tagged nanoconjugates (Figure 3). Next, we assessed if FZD7-NS could inhibit Wnt related genes responsible for cell proliferation, stemness, and metastasis. TNBC cells treated with FZD7-NS exhibited significantly reduced expression of several Wnt pathway markers, with Low FZD7-NS yielding the greatest inhibition, although the difference between Low FZD7-NS and High FZD7-NS was statistically significant only for β-catenin (Figure 4). Given the effective suppression of Wnt signaling in 2D cell culture, we then examined if FZD7-NS could inhibit 3D cancer spheroid formation. FZD7-NS treated spheres had reduced area compared to untreated or PEG-NS treated groups, and samples treated with Low FZD7-NS produced slightly, but statistically significantly smaller spheres compared to those treated with High FZD7-NS when delivered at OD^810 nm^=2 (Figure 5 and Supplemental Figure S8). Spheroid cellular metabolic activity and total cell count in response to FZD7-NS treatment were also significantly reduced at this concentration, with Low FZD7-NS having the greatest effects and being significantly different from High FZD7-NS (Figures 6 and 7).

This study demonstrates the impact of antibody loading density on the ability of antibody-NP conjugates (specifically, FZD7-NS) to target and treat TNBC. The appropriate antibody loading densities for nanoparticle delivery systems are not well-defined in the field. This work revealed that a lower FZD7 antibody loading density of ∼60 per NS (corresponding to ∼670 antibodies per μm^2^) is more beneficial than a higher antibody loading density of ∼170 per NS (∼1848 antibodies per μm^2^) in terms of enabling cell binding and providing a superior therapeutic effect. Although the differences between Low FZD7-NS and High FZD7-NS were modest, they were consistent across numerous assays. We hypothesize that lower loading allows for more extended conformations to increase antibody availability which increases cellular binding, whereas higher loading results in steric hindrance and altered orientation that limits cell binding. Future studies to expand on this work could include a computational approach to investigate how loading density, length of the PEG linker, and the diameter of the core nanoparticle impact antibody conformation and spatial organization on the NP surface. For example, a coarse-grained MARTINI-based model of nanoconjugates incorporating beads for gold, OPSS, PEG, and antibodies could reveal how linker loading density influences antibody conformation and spatial organization. Simulations in GROMACS, coupled with analyses such as radial distribution functions, radius of gyration, and probability distribution functions could reveal whether high antibody loading promotes collapsed conformations and steric crowding that restrict antigen-binding accessibility.

While this study evaluated the role of antibody loading density on cellular interactions of FZD7-NS, future work could extend this further to evaluate the influence of antibody orientation. The conjugation chemistry utilized herein results in random orientation of antibodies on the NS surface such that not all available binding sites may be exposed. Alternatively, FZD7 antibodies could be attached to NS using directional conjugation chemistry.^35^ In prior work, we investigated the influence of E-selectin antibody orientation on NS’ ability to bind endothelial cells (ECs) and thereby block breast cancer cell binding to the ECs.^36^ Similar methods could be used for FZD7-NS, while matching loading density between NS prepared with directional or nondirectional conjugation, to determine how antibody orientation impacts binding to TNBC cells.

To verify the results of the present study are consistent in animal models, future work could transition in vivo to compare the ability of Low FZD7-NS and High FZD7-NS to accumulate in and penetrate throughout TNBC tumors at different stages of disease progression. We have previously shown FZD7-NS can accumulate in TNBC primary tumors and metastatic lesions to reduce disease burden,^15^ but their efficacy as a function of antibody loading density remains to be investigated. In vivo studies should also quantify Wnt target genes in treated tumors to confirm binding levels correlate with downstream signal cascade interference.

Once antibody-NP conjugates have been optimized to have the ideal loading density, it would be interesting to compare these conjugates against NPs that are coated with cell-derived membranes. Numerous studies have shown cell membrane-coated NPs have enhanced cancer cell targeting or tumor delivery compared to their uncoated or PEGylated counterparts;^37–43^ however, direct comparisons between antibody targeting and membrane cloaking strategies are rare. One recent study indicated membrane coated-NPs are superior compared to folic acid-targeted NPs,^37^ but this should be tested for other membrane coatings and antibodies. Since antibodies are specific for a singular receptor whereas membrane proteins can bind multiple receptors on target cells, membrane coatings may be advantageous for targeting due to their ability to overcome heterogeneous receptor expression across individual tumor cells.

In summary, this work shows that a lower density of FZD7 antibodies on the surface of NS is beneficial for enhancing binding to TNBC cells to inhibit oncogenic cell behavior. While the results presented for FZD7-NS are specific to treatment of TNBC, similar findings may apply to antibody-nanoconjugates developed for treatment of other diseases. Our results indicate that antibody loading density is an important parameter that impacts the cellular binding and therapeutic effects of antibody-NP conjugates. Future studies that build on this work will ultimately enable improved decisions regarding the magnitude of antibodies that should be loaded on nanoconjugates to maximize treatment success.

## Supplementary Material



## Notes

Figure S1: correlation function and size distribution by intensity measured by DLS for NS conjugates; Figure S2: multiphoton microscopy images of NS binding to MDA-MB-231 cells; Figure S3: multiphoton microscopy images of NS binding to MCF10A cells; Figure S4: Cy5-NS synthesis and characterization; Figure S5: demonstration of gating parameters of dissociated spheroid cell counting using flow cytometry; Figure S6: NS characterization over 72 h in storage at 4 °C; Figure S7: cellular uptake of Cy5-tagged NS after 24 h incubation quantified via flow cytometry; Figure S8: spheroid growth inhibition 1 week after formation in response to OD = 1 NS treatment; Figure S9: spheroid metabolic activity 1 week after formation in response to OD = 1 NS treatment; Figure S10: cell count of dissociated spheroids 1 week after formation in response to OD = 1 NS treatment; and Table S1: primer sequences used for RT-qPCR studies (PDF)

## ∥

Department of Biomedical Engineering, Brown University, Providence, RI, United States

## ⊥

CPfizer, 401 N Middletown Rd, Pearl River, NY 10965, United States.

## Abbreviations

FZD7: frizzled7
TNBC: triple-negative breast cancer
HER2: human epidermal growth factor receptor 2
NS: nanoshells
APC: adenomatous polyposis coli
GSK-3β: glycogen synthase kinase-3β
CK1α: casein kinase 1 alpha
HauCl4: hydrogen tetrachloroaurate (III) hydrate
NaCl: sodium chloride
OD: optical density
OPSS-PEG-SVA: orthopyridyl disulfide-poly­(ethylene glycol)-succinimidyl valerate
OPSS-PEG-FZD7: orthopyridyl disulfide-poly­(ethylene glycol)-frizzled7 antibody
mPEG-SH: methoxy-poly­(ethylene glycol)-thiol
DLS: dynamic light scattering
TEM: transmission electron microscopy
PBS: phosphate-buffered saline
PBSA: w/v bovine serum albumin in phosphate buffered saline
HRP-AR: horseradish peroxidase antirabbit IgG
TMB Core: 3,3′,5,5′- tetramethylbenzidine
FBS: fetal bovine serum
EDTA: ethylenediaminetetraacetic acid
DPBS: Dulbecco’s phosphate-buffered saline
PBST: phosphate-buffered saline in Tween-20
*K*_D_: effective dissociation constant
Abconc: antibody concentration added to cells
ODraw: initial optical density reading
ODback: background signal optical density
ODhigh: highest saturated optical density
MFI: median fluorescence intensity
RT-qPCR: Real-time reverse transcription quantitative polymerase chain reaction
GUSB: β-glucuronidase
FSC-H: forward scatter height
SSC-H: side scatter height
FSC-A: forward scatter area
