# Supplementary material for: Loading Density Influences the Tumor Cell Targeting and Signaling Inhibition Capabilities of Antibody Nanoconjugates
Source: ACS Omega. 2026 Jan 27;11(5):8836–47. doi: 10.1021/acsomega.5c13065 (PMC12903012; doi:10.1021/acsomega.5c13065)
Supplement: Supplementary file 1 [file ao5c13065_si_001.pdf]

Supporting Information For:

Loading Density Influences the Tumor Cell Targeting and Signaling Inhibition Capabilities of Antibody Nanoconjugates

George C. Kramarenko[1], Carolina Gomez Casas[1,#], Megan N. Dang[1,&], Nikos D. Demetriou[1],

*Emily S. Day[1,2,3]**

[1]Department of Biomedical Engineering, University of Delaware, Newark, DE

[2]Department of Materials Science and Engineering, University of Delaware, Newark, DE

[3]Cawley Center for Translational Cancer Research, Helen F. Graham Cancer Center and Research Institute, Newark, DE

[#]Current address: Department of Biomedical Engineering, Brown University, Providence, RI

[&]Current address: Pfizer, 401 N Middletown Rd, Pearl River, NY 10965

*Corresponding Author: emilyday@udel.edu

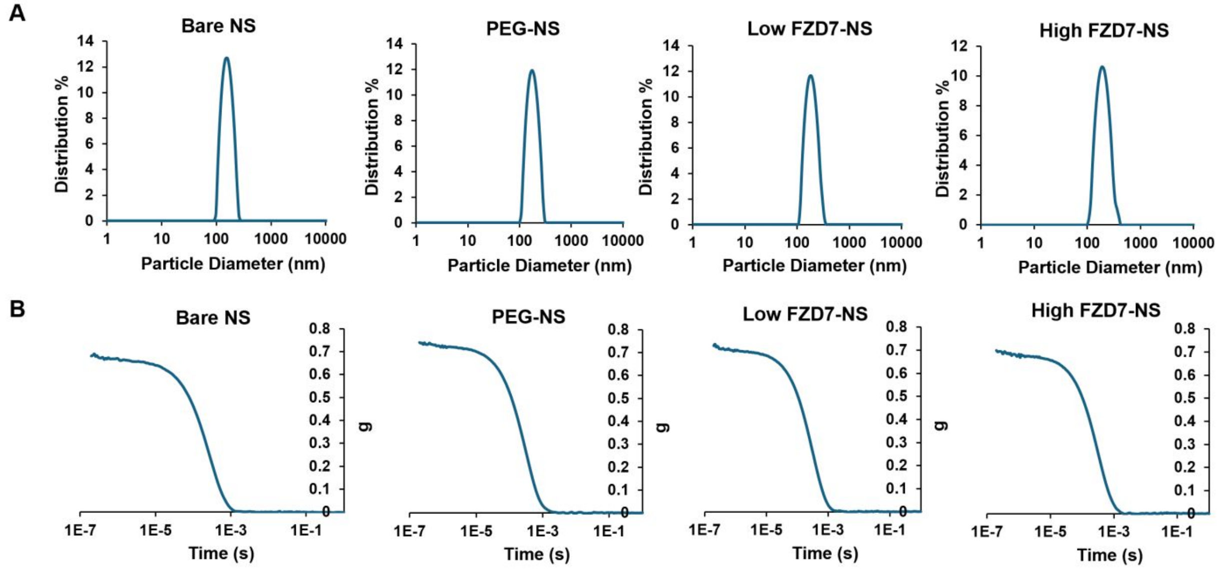

Figure S1. (A) Size distribution and (B) Correlograms showing the field autocorrelation function measured by DLS for samples of Bare NS, PEG-NS, Low FZD7-NS, and High FZD7-NS.

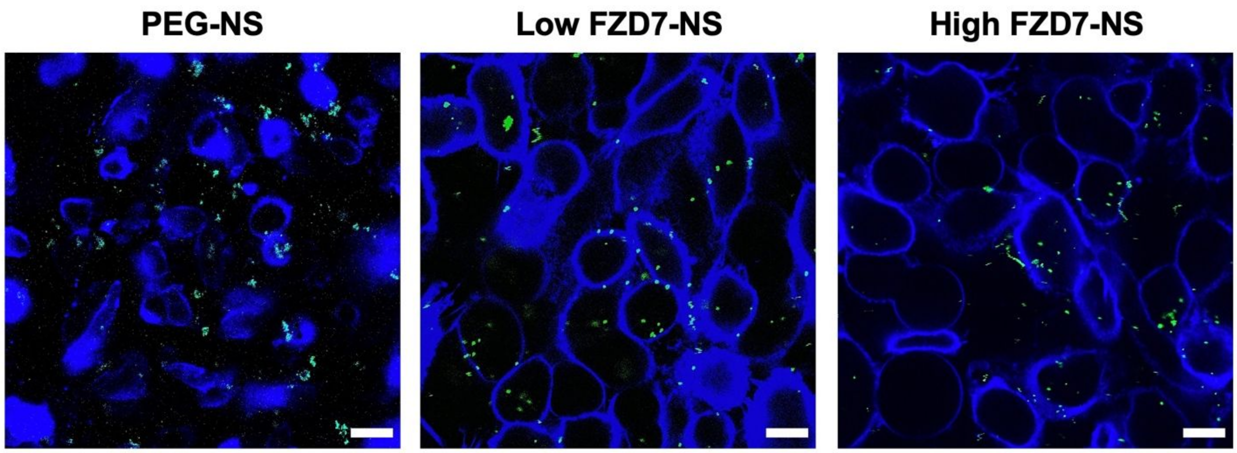

Figure S2. Multiphoton microscopy images of NS (green) binding to MDA-MB-231 cells (blue, labeled with CellVue dye) after 4 hr incubation. Scale bars = 10 μm.

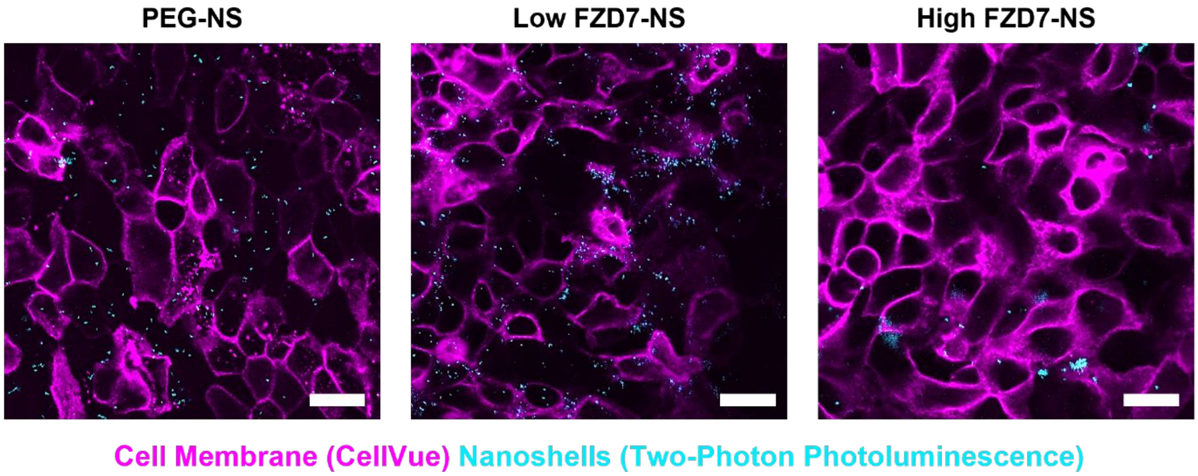

Figure S3. Multiphoton microscopy images of NS binding to MCF10A cells. Cells were treated with NS (cyan) and fixed and stained with CellVue dye (purple). Scale bars = 20 μm.

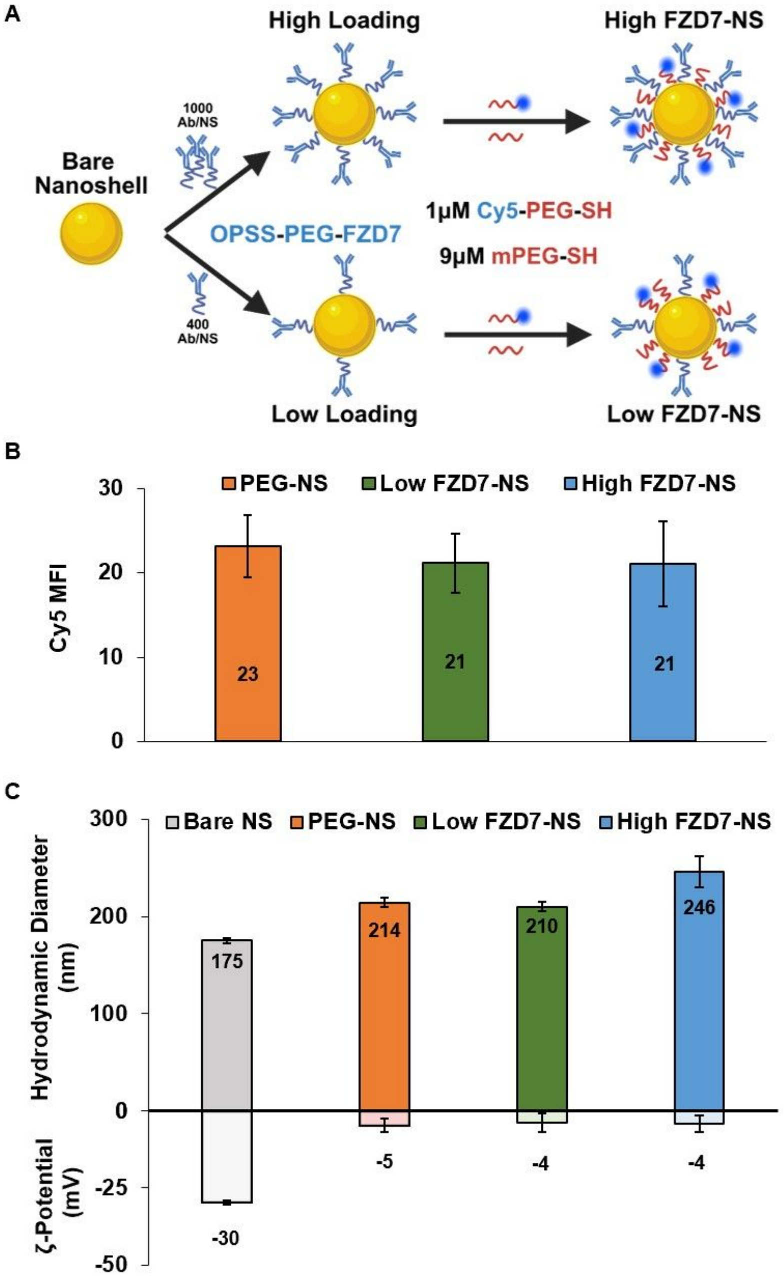

Figure S4. Cy5-NS synthesis and characterization. **(A)** Scheme of Cy5-FZD7-NS conjugation. Created in BioRender. Kramarenko, G. (2026) https://BioRender.com/ul1st6h. **(B)** Quantification of Cy5 fluorescence in NS samples diluted to $OD^{810\ nm}$=1 as measured in a plate reader. MFI=median fluorescence intensity. **(C)** Hydrodynamic diameter and zeta potential measurements

of Bare NS and Cy5-tagged PEG-NS and FZD7-NS. Data represent mean ± standard deviation (n=6).

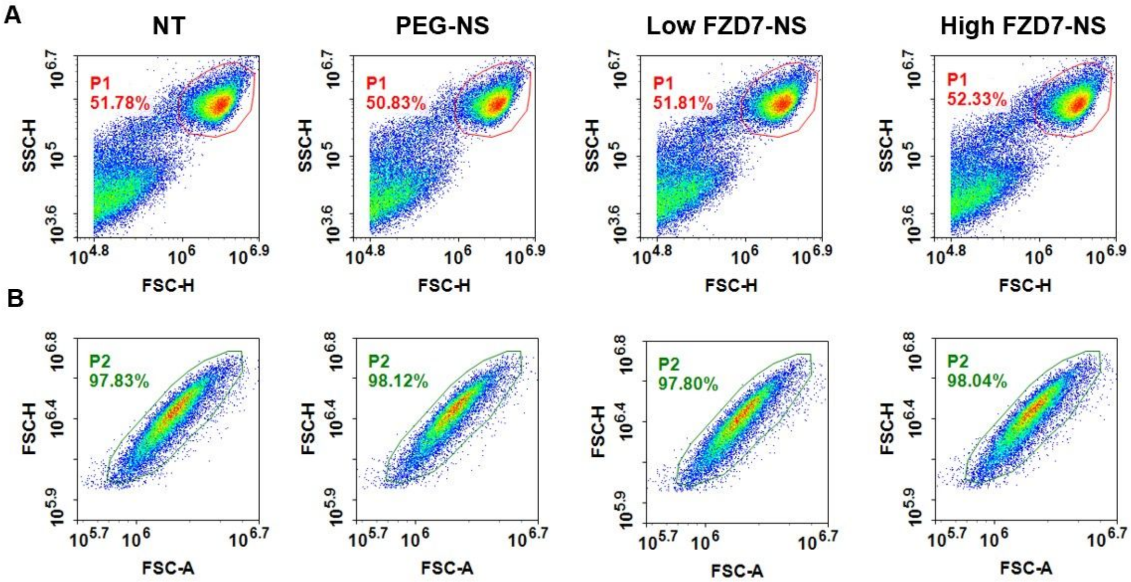

Figure S5. Demonstration of gating parameters for dissociated spheroid cell counting by flow cytometry. **(A)** Initial gate of cell population based on SSC-H vs FSC-H cell sizing. **(B)** Secondary gate of singlet cells based on FSC-H vs FSC-A linearity gating. Cell count was determined from the number of events in the singlet gating parameter.

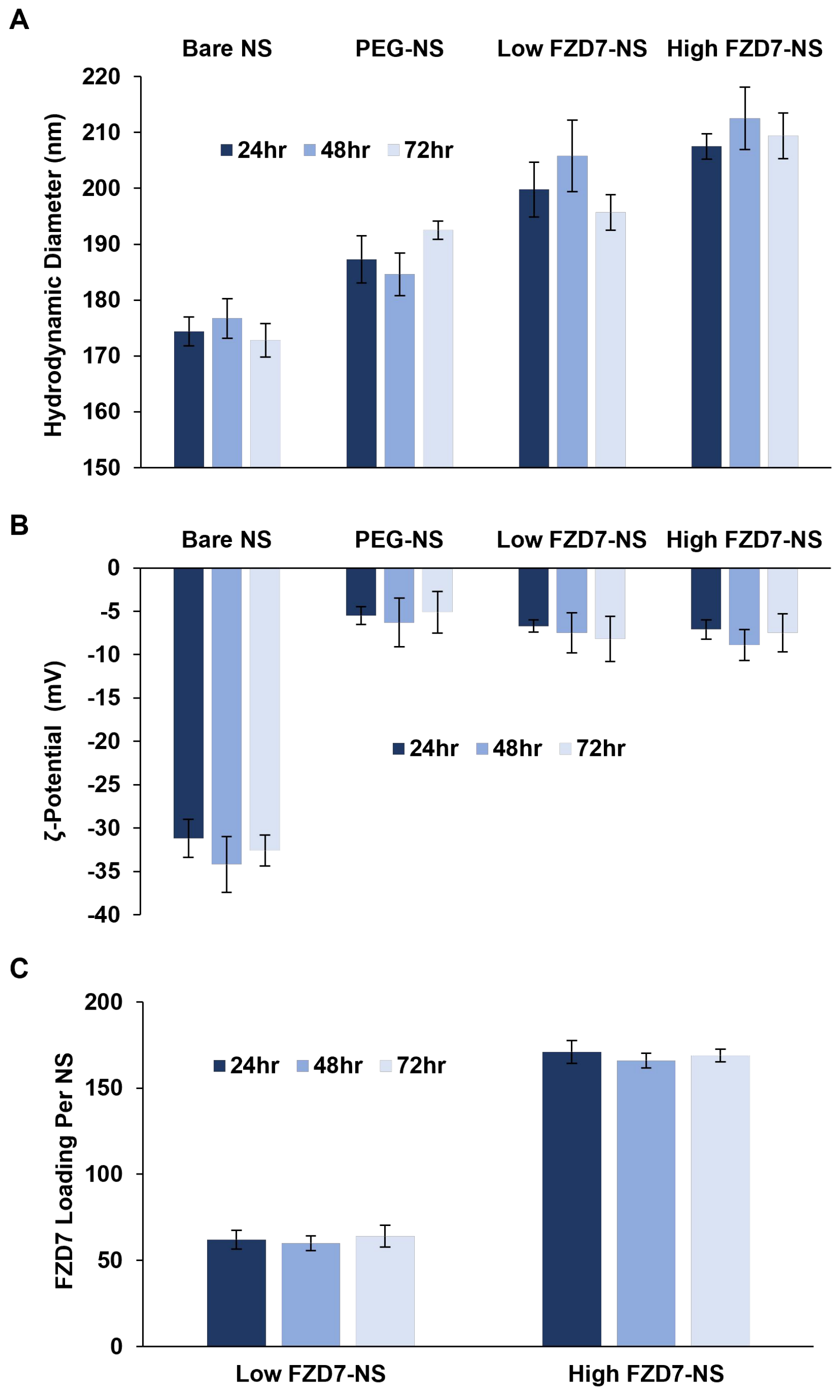

Figure S6. NS characterization over 72 h in storage at 4 ˚C. **(A)** Hydrodynamic diameter, **(B)** Zeta potential, and **(C)** FZD7 antibody loading after 24, 48, or 72 h incubation. Data are mean ± standard deviation (n=6).

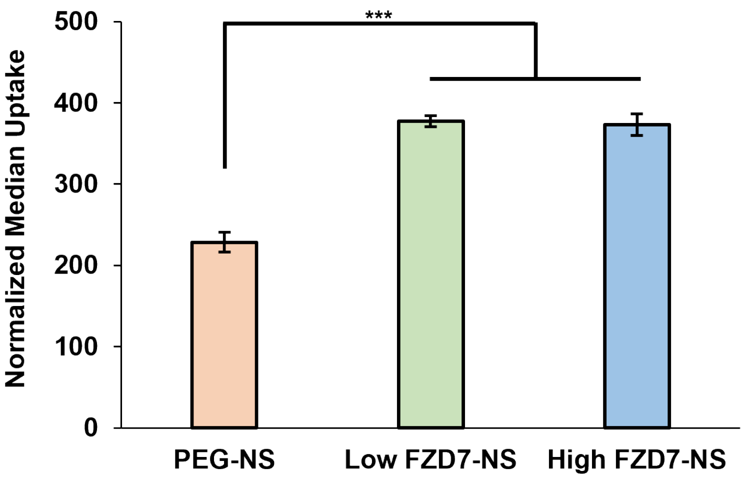

Figure S7. Cellular uptake of Cy5-tagged NS after 24 h incubation at a concentration of OD=1 quantified *via* flow cytometry. Data represent the mean ± standard error of the mean (n=3-4). Statistical analysis by one-way ANOVA with post hoc Tukey-Kramer; ***$p<0.001$ between PEG-NS and both FZD7-NS groups.

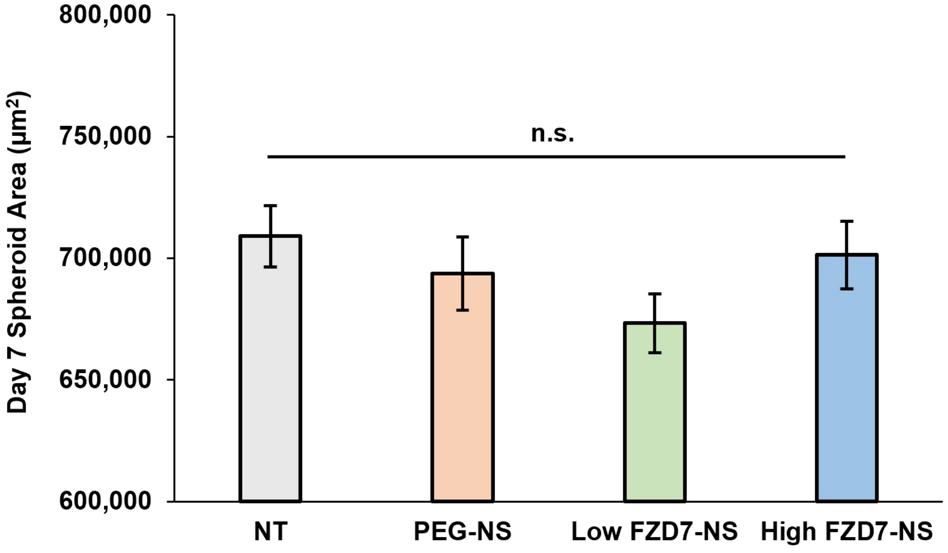

Figure S8. Spheroid growth inhibition one week after formation in response to OD=1 NS treatment. Data show mean ± standard error (n=3). Statistical analysis by one-way ANOVA; n.s.=not significantly different at the 95% confidence level.

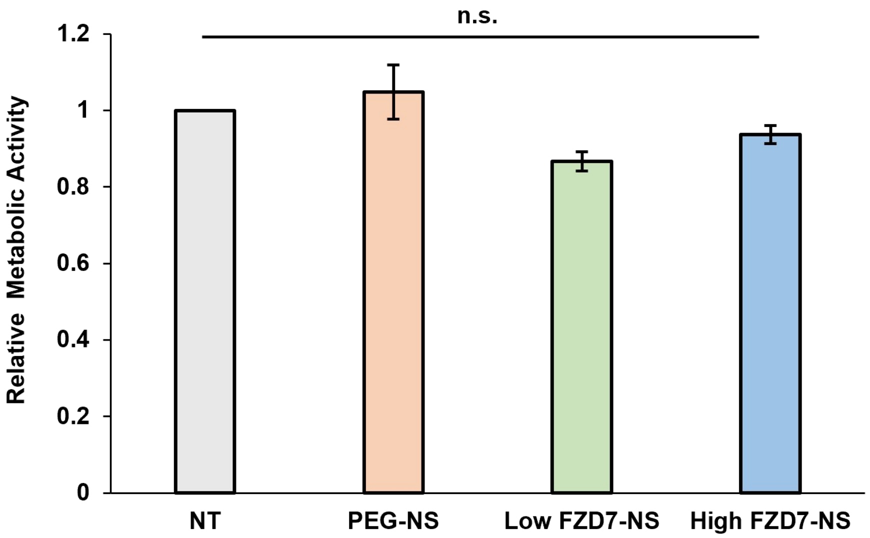

Figure S9. Spheroid metabolic activity one week after formation in response to OD=1 NS treatment measured by AlamarBlue assay. Data are normalized to NT and represent mean ± standard error (n=3). Statistical analysis by one-way ANOVA; n.s.=not significantly different at the 95% confidence level.

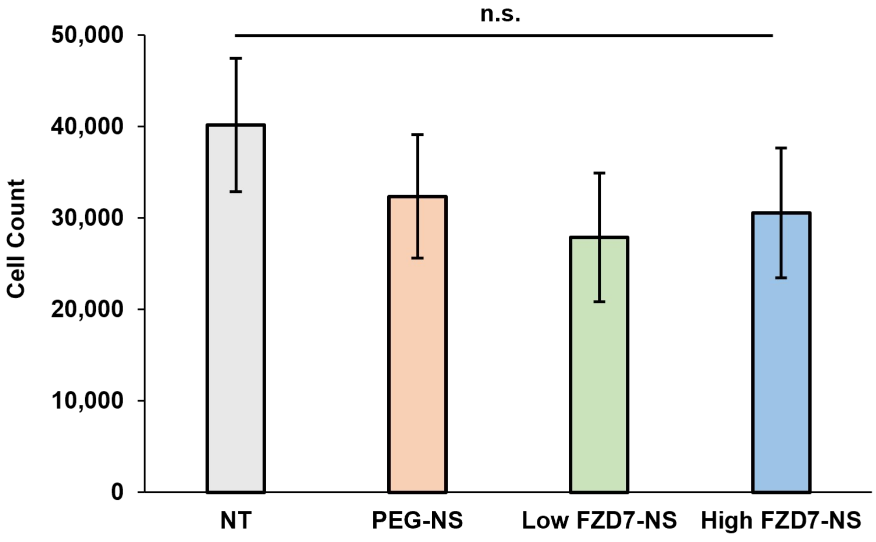

Figure S10. Cell count of dissociated spheroids one week after formation in response to OD=1 NS treatment. Data show mean ± standard error (n=3). Statistical analysis by one-way ANOVA; n.s.=not significantly different at the 95% confidence level.

**GUSB**	TTTTCTTAGCGCCGCAGA<br>GGGCCTGACTCCCACAG
**β-catenin**	GTACGTCCATGGGTGGGACA<br>GGCTCCGGTACAACCTTCAACTA
**Axin2**	TTATGCTTTGCACTACGTCCCTCCA<br>CGCAACATGGTCAACCCTAGAC
**Nanog**	GCAAATGTCTTCTGCTGAGATG<br>GGGCCTGACTCCCACG
**KLF4**	GCCACCCACACTTGTGATTA<br>CAGTCACAGTGGTAAGGTTTCT
**CD44**	GCAGGTATGGGTTCATAGAAGG<br>GGTGTTGGATGTGAGGATGT
**Oct4**	CAGGAGATATGCAAAGCAGAAAC<br>GGCACTGCAGGAACAAATTC

Table S1. Primer sequences used for RT-qPCR studies, listed 5′ to 3′. The effectiveness of FZD7-NS to impact Wnt target gene expression at the mRNA level was assessed for β-catenin, Axin2, Nanog, KLF4, CD44, and Oct4. The housekeeping gene used as a control was GUSB. PCR data was analyzed by the delta delta CT method.
